# Overexpression of miR-451a in sepsis and septic shock patients is involved in the regulation of sepsis-associated cardiac dysfunction and inflammation

**DOI:** 10.1590/1678-4685-GMB-2020-0009

**Published:** 2020-11-16

**Authors:** Heng Wang, Wenjuan Cui, Lujun Qiao, Guoxin Hu

**Affiliations:** 1 Department of Intensive Medicine Department of Intensive Medicine Shengli Oilfield Central Hospital DongyingShandong China Shengli Oilfield Central Hospital, Department of Intensive Medicine, Dongying, Shandong, China.

**Keywords:** MicroRNA-451, sepsis, diagnosis, inflammation, cardiac dysfunction

## Abstract

The purpose of this study was to investigate the expression and clinical value of microRNA-451a (miR-451a) in septic patients and analyze its effect on sepsis-associated cardiac dysfunction and inflammation response. A rat model of sepsis was constructed by cecal ligation and puncture. The expression of miR-451a was measured by quantitative real-time PCR. Receiver operating characteristic (ROC) analysis was used to assess the diagnostic value of serum miR-451a. The cardiac function and inflammatory responses in septic rats were measured to explore the functional role of miR-451a. Serum expression of miR-451a was increased in septic patients compared with healthy controls, and had the ability to distinguish septic patients from healthy volunteers with a sensitivity and specificity of 87.8% and 81.5%, respectively. Elevated serum miR-451a was associated with sepsis severity, as evidenced by the increased expression of miR-451a in septic shock patients and its correlation with key clinical indicators. Significantly upregulated expression of miR-451a was found in septic patients with cardiac dysfunction, and the knockdown of miR-451a in sepsis rats improved cardiac function and inhibited inflammatory responses. All the data revealed that serum miR-451a serves as a candidate diagnostic biomarker of sepsis and a potential parameter to indicate disease severity. The reduction of miR-451a may mitigate sepsis-induced cardiac dysfunction and inflammatory responses.

## Introduction

Sepsis is a systemic inflammatory response syndrome resulting from infection and a leading cause of multiple organ failure and even death (Napolitano, 2018). The pathogenesis of sepsis is characterized by uncontrolled inflammatory responses and immune dysfunction (Rello *et al.,* 2017). Although progress has been made in the treatment and life support for this condition, the mortality of sepsis remains high especially in intensive care units (ICU) (Verdonk *et al.*, 2017). Septic shock (SS) is defined as a subset of sepsis with profound cellular, circulatory and metabolic abnormalities. Patients with SS have a significantly higher mortality rate compared with patients with sepsis alone (Cecconi *et al.*, 2018). Thus, early diagnosis and precise prediction of the onset of SS are important to improve the prognosis of sepsis. Cardiac dysfunction is considered to be a frequent complication of severe sepsis, and is responsible for the deaths occurred in sepsis patients in ICU (Zheng *et al.*, 2017). Therefore, in addition to antibiotics treatments and symptomatic therapeutic methods, such as restoration of blood pressure and systemic perfusion, the strategies to reduce cardiac dysfunction have also received increasing attention (Lv and Wang, 2016).

MicroRNAs (miRNAs) are a group of small noncoding RNAs without protein coding capacity (Reithmair *et al.*, 2017). MiRNAs are characterized by their post-transcriptional regulatory function in gene expression. MiRNAs can directly bind to the 3’-untranslated region (3’-UTR) of targeted messenger RNA (mRNA), leading to the inhibition of gene expression (Sun *et al.*, 2018). In addition, the biological function of miRNAs has been uncovered in various cellular processes, such as proliferation, differentiation, migration, aging and apoptosis (Guo *et al.*, 2018). Multiple miRNAs have been found to be abnormally expressed in human infectious diseases, including sepsis (Benz *et al.*, 2016; Dumache *et al.*, 2015). Some functional miRNAs have been reported to participate in the pathogenesis of sepsis and be related to the onset and development of cardiac dysfunction, such as miR-155 (Zhou *et al.*, 2017) and miR-146a (Gao *et al.*, 2015). A previous study has investigated the circulating miRNAs with aberrant expression in mice subjected to cecal ligation and puncture (CLP), and miR-451a (previously miR-451) was found to be upregulated in an animal model of sepsis (Wu *et al.*, 2013). However, the precise expression patterns of miR-451a in septic patients remain unknown. In addition, the inhibition of miR-451a has been demonstrated to contribute to cardioprotection in several previous publications (Li *et al.*, 2019; Wang *et al.*, 2012). Thus, we wondered whether miR-451a was also involved in the regulation of sepsis-induced cardiac dysfunction.

In this study, the expression of miR-451a was analyzed in septic patients, and its diagnostic value was evaluated. In addition, the effect of miR-451a on sepsis-induced inflammatory responses and cardiac dysfunction was further explored in an animal model of sepsis. The results of this study may provide a novel biomarker of sepsis and a potential therapeutic target for the treatment of sepsis.

## Material and Methods

### Patients and sample collection

A total of 98 septic patients were recruited in this study from the ICU of Shengli Oilfield Central Hospital between 2016 and 2018. The patients were diagnosed following the Surviving Sepsis Campaign: International Guidelines for Management of Severe Sepsis and Septic Shock, 2016 (Rhodes *et al.*, 2017). The exclusion criteria were as follows: 1) less than 18 years old; 2) patients with pregnant or lactating patients; 3) in an immunocompromised state; 4) with positive immunodeficiency virus infection. All of the patients were further grouped into non-SS group (n = 70) and SS group (n = 28) based on the occurrence of SS according to the Third International Consensus Definitions for Sepsis and Septic Shock (Singer *et al.*, 2016). According to the heart function monitor results, 59 septic patients had cardiac dysfunction, including 19 SS patients and 40 non-SS patients. In addition, 65 healthy volunteers were enrolled to serve as a control group. Venous blood was collected from the participants and serum samples were extracted by centrifugation. The demographic and clinical characteristics of the study population, including age, gender, body mass index (BMI), albumin, serum creatinine (Scr), white blood cell (WBC), C-reactive protein (CRP), procalcitonin (PCT), Acute Physiology and Chronic Health Evaluation II (APACHE II) score and Sequential Organ Failure Assessment (SOFA) score, were summarized in Table 1. This study was performed with the approval of the Ethics Committee of Shengli Oilfield Central Hospital, and written informed consent was obtained from each patient.

**Table 1:** Baseline characteristics of sepsis patients and healthy volunteers.

Features	Healthy controls	Sepsis patients	*P* value
	(n = 65)	(n = 98)	
Age	58.43 ± 6.67	57.35 ± 7.33	0.340
(mean ± SD, year)			
Gender	38/27	56/42	0.867
(male/female)			
BMI	21.30 ± 2.08	21.38 ± 2.11	0.816
(mean ± SD, kg/m^2^)			
Scr	1.10 ± 0.29	1.83 ± 0.23	< 0.001
(mean ± SD, mg/dL)			
Albumin	39.06 ± 3.48	24.67 ± 2.90	< 0.001
(mean ± SD, g/L)			
WBC	8.04 ± 0.88	18.94 ± 3.55	< 0.001
(mean ± SD, 10^9^/L)			
CRP	6.04 ± 1.20	97.65 ± 19.77	< 0.001
(mean ± SD, mg/L)			
PCT	0.04 ± 0.02	13.07 ± 3.74	< 0.001
(mean ± SD, ng/mL)			
APACHE II score	-	11.57 ± 2.66	-
(mean ± SD)			
SOFA score	-	5.60 ± 1.55	-
(mean ± SD)			

### Animal grouping and sepsis animal model

Male Sprague–Dawley (SD) rats (weighting 250 – 300 g) were purchased from the Laboratory Animal Center of Nanjing Medical University (Nanjing, Jiangsu Province, China) and grouped into four groups, including sham group (n = 10), sepsis model group (n = 8), miR-451a negative control (NC) group (n = 9) and miR-451a antagomir group (n = 10). Each group included at least eight viable individuals. Rats in the sepsis model group were subjected to CLP to induce sepsis condition as previously described (Dejager *et al.*, 2011). Briefly, after the anesthesia with sodium pentobarbital (50 mg/kg, Sigma, St. Louis, MO, USA), a midline incision was conducted on the rats’ anterior abdomen, then the cecum was ligated at its position of 30%. The cecum was punctured twice before closing the abdominal cavity, and the fecal material was extruded. The rats in the miR-451a NC group and miR-451a antagomir group were intravenously injected with miR-451a NC sequence (10 μg; 5’-UUGUACUACAAAAGUACUG-3’) or miR-451a antagomir (10 μg; 5’-AACUCAGUAAUGGUAACGGUUU-3’; GenePharma, Shanghai, China), respectively, at 24 h prior to the surgery. Rats in the sham group received the same surgical procedure without ligation and puncture. After the surgery, all of the rats were injected with 1 mL of normal saline for resuscitation. All rats were killed 12 hours postoperatively with an overdose of a general anesthetic (thiopental sodium, 50 mg/kg), and the myocardial tissues were collected quickly.

### RNA extraction and quantitative real-time PCR (qRT-PCR)

Total RNA was extracted from serum of patients and serum and myocardial tissues of rats using Trizol reagent (Life Technologies Corporation, Carlsbad, CA, USA). cDNA was obtained by reveres transcription from RNA by using a TaqMan miRNA reverse transcription kit (Applied Biosystems, Foster City, CA, USA). The relative expression of miR-451a was analyzed using quantitative PCR (qPCR) with a One Step SYBR® PrimeScript®PLUS RT-RNA PCR kit (TaKaRa Biotechnology, Dalian, China), and the final expression values were calculated using the comparative delta CT (2^−Ä^^D^^Ct^) method, with normalization to U6.

### Cardiac function and blood cytokines analyses

The cardiac function and inflammatory responses of rats in different groups were analyzed. As previously described (Chen *et al.*, 2018), after the rats were anesthetized, the catheter was inserted into the left ventricle through the right common artery. Then the cardiac function of rats was analyzed by the MFLab 3.01 package in FDP-1 HRV & BRS analysis system (Shanghai Jialong, Shanghai, China), the left ventricular peak pressure (LVPP), left ventricular end diastolic pressure (LVEDP) and maximum rate of rise/fall of left ventricle pressure (± dp/dt_max_) were examined. After collecting blood samples were collected from rats in each group, an enzyme-linked immunosorbent assay (ELISA) was used to measure the levels of cardiac function biomarkers, including cardiac troponin I (cTnI) and creative kinase isoenzyme MB (CK-MB), and pro-inflammatory cytokines, including tumor necrosis factor alpha (TNF-α), interleukin 6 (IL-6) and IL-1β.

### Statistical analysis

Data obtained from this study were expressed as mean ± SD and were analyzed using the SPSS version 18.0 software (SPSS Inc., Chicago, IL) and GraphPad Prism 5.0 software (GraphPad Software, Inc., USA). Differences between groups were analyzed using Student's *t* test, Chi-square test or one-way ANOVA. Correlation analysis was performed using Pearson correlation coefficient. Receiver operating characteristic (ROC) analysis was performed to evaluate the diagnostic value of serum miR-451a in septic patients. A *P* value of less than 0.05 was considered to be statistically significant.

## Results

### Baseline characteristics of the study population

The demographic and clinical characteristics of the study population were listed in Table 1. The results indicated that there were no differences in age, gender and BMI between the septic patients and healthy controls (all *P* > 0.05). Compared with the healthy controls, septic patients had higher serum levels of Scr, WBC, CRP and PCT and lower albumin concentrations (all *P* < 0.001). This study evaluated the APACHE II score and SOFA score to determine the severity of sepsis in patients, and the APACHE II score was 11.57 ± 2.66 and SOFA score was 5.60 ± 1.55.

### Upregulated expression of miR-451a in sepsis

**Figure 1:**
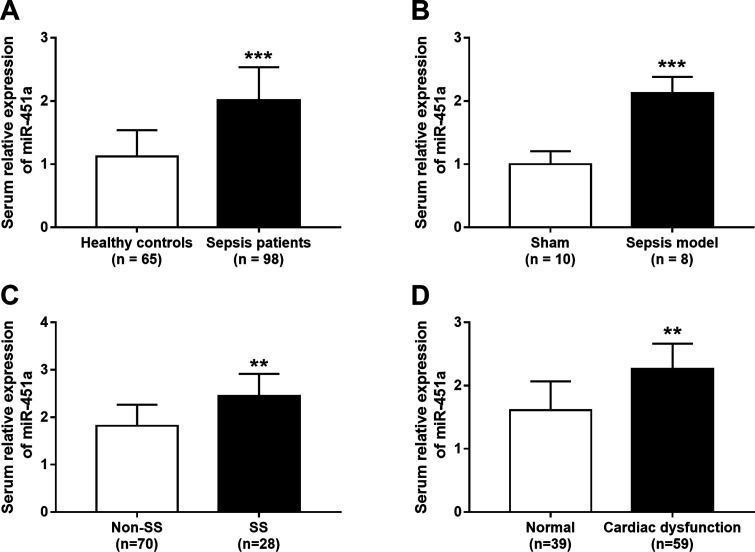
Serum expression of miR-451a in sepsis patients and animals. A. Serum miR-451a expression levels were measured in 65 healthy controls and 98 septic patients. MiR-451a was significantly increased in sepsis patients compared with that in the healthy conrols. B. In septic rats model, serum miR-451a expression was significantly higher than that in sham group. n = 10 rats in sham group, n = 8 rats in septic group. C. Total 98 septic patients were divided into non-SS group and SS group, qRT-PCR results indicated that serum miR-451a expression was increased in SS cases compared with non-SS cases. D. According to heart function monitor results, 59 septic patients were determined with cardiac dysfunction, while other 39 cases without cardiac dysfunction were identified as normal group. Serum miR-451a expression was increased in sepsis patients with cardiac dysfunction compared with the cases with normal cardiac function. **P < 0.01, **P < 0.001.

According to qRT-PCR, the expression of miR-451a in the study population was examined. As shown in Figure 1A, serum expression of miR-451a was significantly increased in septic patients compared with healthy controls (*P* < 0.001). Similarly, the elevated expression of miR-451a was also observed in the sepsis rats (*P* < 0.001, Figure 1B), which was generated by CLP. The 98 septic patients included 28 SS cases, and the expression data showed that miR-451a expression was higher in SS patients than in the the non-SS cases (*P* < 0.01, Figure 1C), indicating that miR-451a might be involved in the severity of sepsis. Furthermore, the cardiac function in the patients was evaluated, and 59 cases with cardiac dysfunction were found among the 98 septic patients. Notably, the patients with cardiac dysfunction had the higher miR-451a expression levels than those with normal cardiac function (*P* < 0.01, Figure 1D)

### Relationship of miR-451a expression with clinical characteristics of septic patients

To further explore the potential role of miR-451a in the development of sepsis, the relationship between miR-451a and patients’ clinical data was assessed. The results listed in Table 2 indicated that serum miR-451a was positively correlated with Scr, WBC, PCT, CRP, APACHE II score and SOFA score (all *P* < 0.05), but had no significant correlation with age, gender, BMI and albumin (all *P* > 0.05), which indicated that miR-451a might be involved in the progression of sepsis and associated with disease severity.

**Table 2:** Correlation of miR-451a with clinical characteristics of sepsis patients.

Parameters	miR-451a expression	
	Correlation coefficient (*r*)	*P* value
Age	0.113	0.534
Gender	0.057	0.629
BMI	0.145	0.582
Scr	0.233	0.045
Albumin	-0.134	0.089
WBC	0.548	< 0.001
CRP	0.519	< 0.001
PCT	0.602	< 0.001
APACHE II score	0.621	< 0.001
SOFA score	0.647	< 0.001

### Diagnostic performance of miR-451a in patients with sepsis

**Figure 2:**
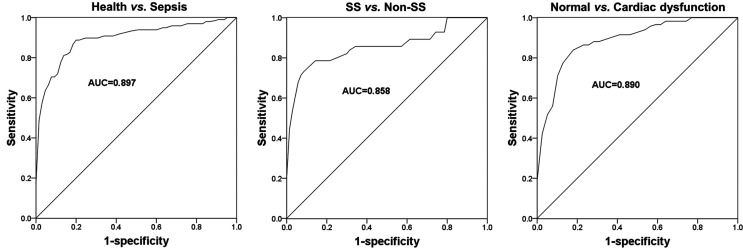
ROC curves in sepsis patients based on serum miR-451a levels. A. A ROC curve to distinguish septic patients from healthy volunteers. B. A ROC curve to distinguish SS cases from non-SS cases. C. A ROC curve to distinguish septic patients with cardiac dysfunction from the cases with normal cardiac function. AUC, area under the curve.

Considering the significantly increased expression of miR-451a in sepsis patients, an ROC curve based on serum miR-451a was constructed to evaluate the diagnostic potential of miR-451a. As shown in Figure 2A, the area under the curve (AUC) was 0.897, with a sensitivity and specificity of 87.8% and 81.5%, respectively, at a cutoff value of 1.465, suggesting the diagnostic accuracy of miR-451a to distinguish septic patients from healthy volunteers. In addition, this study further evaluated the ability of miR-451a to differentiate SS patients from non-SS patients. The ROC curve shown in Figure 2B indicated that miR-451a could screen SS cases from non-SS patients, yielding an AUC of 0.858 (sensitivity: 75.0%, specificity: 91.4%, cutoff value: 2.255). Furthermore, given the higher miR-451a expression in patients with cardiac dysfunction, a ROC curve based on serum miR-451a in patients with different cardiac function status was plotted. As shown in Figure 2C, serum miR-451a might have a relatively good clinical value to predict the occurrence of cardiac dysfunction in septic patients, with an AUC of 0.890, a sensitivity of 86.4% and specificity of 82.1% at the cutoff value of 1.955.

### Inhibition of miR-451a suppresses cardiac dysfunction in septic animals

**Figure 3:**
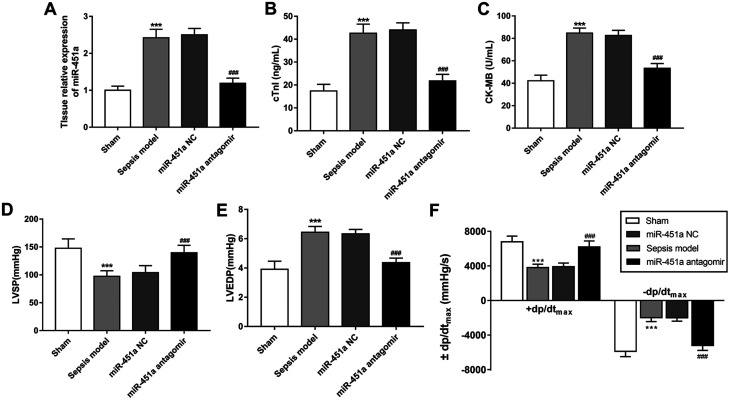
Effects of miR-451a on cardiac function in sepsis rats. A. The increased miR-451 in myocardial tissues in sepsis rats was inhibited by the miR-451a antagomir. B and C. Levels of cardiac function markers, including cTnI (B) and CK-MB (C), in septic rats with downregulated miR-451a. D-F. Effects of miR-451a on the hemodynamic parameters, including LVSP (D), LVEDP (E) and æ dp/dtmax (F) in sepsis rats. n = 10 rats in sham group, n = 8 rats in sepsis group, n = 9 rats in miR-NC group, and n = 10 in miR-451a antagomir group. The data was shown as mean and SD. ***P < 0.001, compared with sham group; ###P < 0.001, compared with sepsis model group.

To further understand the effect of miR-451a on cardiac dysfunction in sepsis, this study constructed a sepsis animal model and the expression of miR-451a was regulated by miR-451a antagomir transfection. The data shown in Figure 3A indicated that the expression of miR-451a was markedly downregulated by the miR-451a antagomir (*P* < 0.001). In the CLP rat model, the LVSP and +dp/dt_max_ were decreased, while the LVEDP, -dp/dt_max_, cTnI and CK-MB were increased (all *P* < 0.01, Figure 3B-F), suggesting that the cardiac function of septic rats was disordered. Notably, the impaired cardiac function was significantly improved by the reduction of miR-451a, which was indicated by the increased LVSP and +dp/dt_max_ and decreased LVEDP, -dp/dt_max_, cTnI and CK-MB (all *P* < 0.001, Figure 3B-F).

### Knockdown of miR-451a inhibits inflammatory responses in septic rats

**Figure 4:**
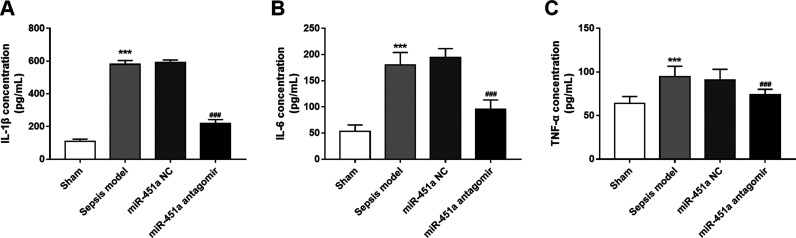
Effects of miR-451a on inflammatory response in sepsis rats. A. Regulatory effect of miR-451a on serum levels of IL-1b. B. Regulatory effect of miR-451a on serum levels of IL-6. C. Regulatory effect of miR-451a on serum levels of TNF-a. n = 10 rats in sham group, n = 8 rats in sepsis group, n = 9 rats in miR-NC group, and n = 10 in miR-451a antagomir group. The data was shown as mean and SD. ***P < 0.001, compared with sham group; ###P < 0.001, compared with sepsis model group.

The inflammatory responses in the septic rats were further analyzed. The results shown in Figure 4 indicated that the inflammatory responses were enhanced in the sepsis model compared with the sham rats, as evidenced by the significantly increased IL-1β, IL-6 and TNF-α levels (all *P* < 0.001). After knockdown of miR-451a, the increased levels of IL-1β, IL-6 and TNF-α were reduced significantly (all P < 0.01), indicating that the inhibition of miR-451a in sepsis led to suppressed inflammation.

## Discussion

Numerous miRNAs with aberrant expression have been identified in various human diseases, including infectious diseases, such as sepsis (Pfeiffer *et al.*, 2017; Yao *et al.*, 2018). These miRNAs are deregulated during disease development and involved in the regulation of disease progression (Sheng *et al.*, 2017). For example, miR-1247 was found to be downregulated in infantile pneumonia patients and its overexpression might be a novel therapeutic strategy by alleviating lipopolysaccharide (LPS)-induced lung injury (Guo and Cheng, 2018). The upregulated expression of miR-155 in active tuberculosis patients could inhibit monocyte apoptosis during the pathogenesis of tuberculosis (Huang *et al.*, 2015). In patients with sepsis, some miRNAs with abnormal expression have also been identified, such as miR-150 (Ma *et al.*, 2018), miR-21-3p (Wang *et al.*, 2016) and miR-375 (Sheng *et al.*, 2017), which have been reported to participate in the progression of sepsis. In this study, we found that miR-451a expression was significantly increased in sepsis patients and sepsis animals, which was consistent with the results in a previous study that also found the overexpression of miR-451 in sepsis mice (Wu *et al.*, 2013). Additionally, a similar result was also observed in neonatal septic patients, suggesting that serum miR-451 is upregulated significantly in neonatal septic patients compared with control subjects (Benz *et al.*, 2016; Wang *et al.*, 2015). The expression results indicated that miR-451a might play a potential role in the development of sepsis.

Serum miRNAs have been considered to be a group of good diagnostic tools for their stability in circulating system and their markedly aberrant expression patterns (Benz *et al.*, 2016). Considering the significant increase in the expression of miR-451a in septic patients, this study further evaluated its diagnostic value by ROC analysis. The AUC results suggested that miR-451a had a high accuracy to distinguish septic patients from healthy volunteers. Deregulated miR-451a in other diseases has been proposed as a potential diagnostic biomarker. In gastric cancer, the decreased expression of miR-451 in gastric cancer tissues was demonstrated to be a diagnostic and prognostic biomarker (Shen *et al.*, 2017). Plasma miR-451 combined with echocardiography has been reported to have good diagnostic value when diagnosing pulmonary hypertension (Song *et al.*, 2018). The previous data combined with our ROC results led us to conclude that the increased serum miR-451a might serve as a candidate diagnostic biomarker of sepsis.

The changes in the levels of PCT, CRP and WBC are established indicators and are widely used in clinical practices that reflect the clinical status and prognosis of sepsis (Yang *et al.*, 2016). In this study, significantly elevated levels of PCT, CRP and WBC were detected in septic patients, and their levels were found to be positively correlated with serum expression of miR-451a, which suggested that miR-451a might be involved in the development of sepsis. In addition, the APACHE II score and SOFA score were measured to reflect the severity of septic patients, and the positive correlations of miR-451a with these two score were also found, indicating that the serum elevated miR-451a was associated with disease severity in septic patients. It is known that SS may develop as the disease progresses in sepsis (Armstrong *et al.*, 2017). The expression changes of miR-451a between the non-SS patients and the SS patients were compared, and showed that miR-451a expression was higher in the SS patients. Additionally, the ROC analysis results revealed that elevated serum miR-451a expression could distinguish SS patients from non-SS patients with relatively high accuracy. Thus, we considered that the serum miR-451a might be a potential molecule to indicate the severity of sepsis.

Sepsis-induced dysfunction in multiple organs is the leading cause of death, and cardiac dysfunction frequently occurs in septic patients and significantly promotes disease mortality rates (Havaldar, 2018). Currently, several miRNAs have been reported to be related to the development of cardiac dysfunction in sepsis. For example, the increased miR-155 expression in septic mice has been found to attenuate cardiac dysfunction and improve disease survival (Zhou *et al.*, 2017). miR-146a could mitigate myocardial injury and inflammatory cytokine production in sepsis (Gao *et al.*, 2015). The elevated expression of miR-21-3p in sepsis has been demonstrated to be associated with sepsis-induced cardiac dysfunction (Wang *et al.*, 2016). In this study, compared with that of the septic patients with normal cardiac function, the expression of miR-451a in the septic patients with cardiac dysfunction was significantly higher, and this elevation was shown to be high accurate in distinguishing cases with cardiac dysfunction from cases with the normal cardiac function, which indicated that miR-451a was associated with sepsis-induced cardiac dysfunction and might have the potential to predict the onset of cardiac dysfunction in sepsis patients. To further verify the effect of miR-451a on sepsis-related cardiac dysfunction, the expression of miR-451a was downregulated in a rat model of sepsis. As previously described, rats were subjected to CLP to induce sepsis condition. Consistent with the previous study, cardiac function of rats was impaired after CLP (Guo *et al.*, 2019). Moreover, after the knockdown of miR-451a, the impaired cardiac function in sepsis rats, which evidenced by serum cardiac function biomarkers and hemodynamic index, was significantly improved. The results regarding cardiac function in this study suggested that the inhibition of miR-451a might protect against the cardiac dysfunction in sepsis. However, the current study only focused on the role of miR-451a in sepsis-induced cardiac dysfunction. It would be interesting to investigate the half-life and the pharmacokinetics of miR-451a in the heart and circulation, which will be beneficial for the understanding the mechanism and the applications of targeted therapy involving miRNAs in sepsis.

Uncontrolled inflammatory responses are important in the pathogenesis of sepsis and can aggravate the disease progression and also the development of multifunctional organ failure, including cardiac injury. The abnormal expression of miRNAs has been reported to be involved in the regulation of inflammatory response in several human disease, including sepsis. For example, miR-495 has been identified to be downregulated in the serum of sepsis patients, and the in vitro study further confirmed that overexpression of miR-495 can alleviate CLP induced inflammatory response (Guo *et al.*, 2019). In the present study, we found that the enhanced inflammatory response was remarkably reversed by the reduction of miR-451a in our constructed septic rat model, as shown by the decreases in pro-inflammatory cytokine levels. Thus, we deduced that the improved cardiac function in septic rats by the knockdown of miR-451a might be achieved by inhibiting inflammatory responses. Although this study provided evidence for the important regulatory effect of miR-451a on sepsis-induced cardiac dysfunction, the underlying molecular mechanisms remain unclear and warrant further investigation. MiRNAs function primarily as post-transcriptional negative regulators of gene expression via binding to their mRNA targets. As previous studies reported, several target genes have been shown to be involved in the role of miR-451a in the progression of several diseases (Guo *et al.*, 2017; Weng *et al.*, 2019). Calcium-binding protein 39 (Cab-39) is a scaffold protein of liver kinase B1 (LKB1), and was identified as a direct target gene of miR-451a in several diseases (Guo *et al.*, 2017; Nan *et al.*, 2018). Cab-39 can activate the AMPKα signaling pathway, which has been suggested to exert a cardioprotective role (Konishi *et al.*, 2011; Wang *et al.*, 2017). Additionally, AMPKα has been suggested to improve cardiac function in mice with sepsis, and depletion of AMPKα contributes to the development of sepsis (Huang *et al.*, 2018; Song *et al.*, 2020). Accordingly, we speculated that downregulation of miR-451a may alleviate cardiac dysfunction in septic rats via targeting Cab-39 and regulating AMPKα signaling. However, further studies are needed to verify our hypothesis.

In conclusion, serum miR-451a expression was elevated and correlated with disease severity in septic patients and associated with the onset of cardiac dysfunction. The reduction of miR-451a could alleviate cardiac dysfunction and inflammatory responses in septic rats. The results of this study provide a novel serum diagnostic biomarker of sepsis and a theoretical basis for studying the mechanism of sepsis induced cardiac dysfunction.

## References

[B1] Armstrong BA, Betzold RD and May AK (2017) Sepsis and septic shock strategies. Surg Clin North Am 97:1339-1379. 10.1016/j.suc.2017.07.003 29132513

[B2] Benz F, Roy S, Trautwein C, Roderburg C and Luedde T (2016) Circulating MicroRNAs as biomarkers for sepsis. Int J Mol Sci 17:78. 10.3390/ijms17010078 PMC4730322 26761003

[B3] Cecconi M, Evans L, Levy M and Rhodes A (2018) Sepsis and septic shock. Lancet 392:75-87. 10.1016/S0140-6736(18)30696-2 29937192

[B4] Chen H, Wang X, Yan X, Cheng X, He X and Zheng W (2018) LncRNA MALAT1 regulates sepsis-induced cardiac inflammation and dysfunction via interaction with miR-125b and p38 MAPK/NFκB. Int Immunopharmacol 55:69-76. 10.1016/j.intimp.2017.11.038 29227823

[B5] Dejager L, Pinheiro I, Dejonckheere E and Libert C (2011) Cecal ligation and puncture: the gold standard model for polymicrobial sepsis? Trends Microbiol 19:198-208. 10.1016/j.tim.2011.01.001 21296575

[B6] Dumache R, Rogobete AF, Bedreag OH, Sarandan M, Cradigati AC, Papurica M, Dumbuleu CM, Nartita R and Sandesc D (2015) Use of miRNAs as biomarkers in sepsis. Anal Cell Pathol (Amst) 2015:186716. 10.1155/2015/186716 PMC4499375 26221578

[B7] Gao M, Wang X, Zhang X, Ha T, Ma H, Liu L, Kalbfleisch JH, Gao X, Kao RL, Williams DL *et al.* (2015) Attenuation of cardiac dysfunction in polymicrobial sepsis by microRNA-146a is mediated via targeting of IRAK1 and TRAF6 expression. J Immunol 195:672-682. 10.4049/jimmunol.1403155 PMC4490963 26048146

[B8] Guo H, Tang L, Xu J, Lin C, Ling X, Lu C and Liu Z (2019) MicroRNA-495 serves as a diagnostic biomarker in patients with sepsis and regulates sepsis-induced inflammation and cardiac dysfunction. Eur J Med Res 24:37. 10.1186/s40001-019-0396-3 PMC6878688 31771650

[B9] Guo J and Cheng Y (2018) MicroRNA-1247 inhibits lipopolysaccharides-induced acute pneumonia in A549 cells via targeting CC chemokine ligand 16. Biomed Pharmacother 104:60-68. 10.1016/j.biopha.2018.05.012 29768218

[B10] Guo J, Liu Q, Li Z, Guo H, Bai C and Wang F (2018) miR-222-3p promotes osteosarcoma cell migration and invasion through targeting TIMP3. Onco Targets Ther 11:8643-8653. 10.2147/OTT.S175745 PMC6284535 30584323

[B11] Guo R, Gu J, Zhang Z, Wang Y and Gu C (2017) miR-451 promotes cell proliferation and metastasis in pancreatic cancer through targeting CAB39. Biomed Res Int 2017:2381482. 10.1155/2017/2381482 PMC5288510 28197410

[B12] Havaldar AA (2018) Evaluation of sepsis induced cardiac dysfunction as a predictor of mortality. Cardiovasc Ultrasound 16:31. 10.1186/s12947-018-0149-4 PMC6267025 30501628

[B13] Huang J, Jiao J, Xu W, Zhao H, Zhang C, Shi Y and Xiao Z (2015) miR-155 is upregulated in patients with active tuberculosis and inhibits apoptosis of monocytes by targeting FOXO3. Mol Med Rep 12:7102-7108. 10.3892/mmr.2015.4250 26324048

[B14] Huang J, Liu K, Zhu S, Xie M, Kang R, Cao L and Tang D (2018) AMPK regulates immunometabolism in sepsis. Brain Behav Immun 72:89-100. 10.1016/j.bbi.2017.11.003 29109024

[B15] Konishi M, Haraguchi G, Ohigashi H, Ishihara T, Saito K, Nakano Y and Isobe M (2011) Adiponectin protects against doxorubicin-induced cardiomyopathy by anti-apoptotic effects through AMPK up-regulation. Cardiovasc Res 89:309-319. 10.1093/cvr/cvq335 20978005

[B16] Li J, Wan W, Chen T, Tong S, Jiang X and Liu W (2019) miR-451 Silencing inhibited doxorubicin exposure-induced cardiotoxicity in mice. Biomed Res Int 2019:1528278. 10.1155/2019/1528278 PMC6637715 31355248

[B17] Lv X and Wang H (2016) Pathophysiology of sepsis-induced myocardial dysfunction. Mil Med Res 3:30. 10.1186/s40779-016-0099-9 PMC5037896 27708836

[B18] Ma Y, Liu Y, Hou H, Yao Y and Meng H (2018) miR-150 predicts survival in patients with sepsis and inhibits LPS-induced inflammatory factors and apoptosis by targeting NF-kappaB1 in human umbilical vein endothelial cells. Biochem Biophys Res Commun 500:828-837. 10.1016/j.bbrc.2018.04.168 29689269

[B19] Nan Y, Guo H, Guo L, Wang L, Ren B, Yu K, Huang Q and Zhong Y (2018) miRNA-451 inhibits glioma cell proliferation and invasion through the mTOR/HIF-1α/VEGF signaling pathway by targeting CAB39. Hum Gene Ther Clin Dev 29:156-166. 10.1089/humc.2018.133 30180756

[B20] Napolitano LM (2018) Sepsis 2018: definitions and guideline changes. Surg Infect (Larchmt) 19:117-125. 10.1089/sur.2017.278 29447109

[B21] Pfeiffer D, Rossmanith E, Lang I and Falkenhagen D (2017) miR-146a, miR-146b, and miR-155 increase expression of IL-6 and IL-8 and support HSP10 in an In vitro sepsis model. PLoS One 12:e0179850. 10.1371/journal.pone.0179850 PMC5491059 28662100

[B22] Reithmair M, Buschmann D, Marte M, Kirchner B, Hagl D, Kaufmann I, Pfob M, Chouker A, Steinlein OK, Pfaffl MW *et al.* (2017) Cellular and extracellular miRNAs are blood-compartment-specific diagnostic targets in sepsis. J Cell Mol Med 21:2403-2411. 10.1111/jcmm.13162 PMC5618677 28382754

[B23] Rello J, Valenzuela-Sanchez F, Ruiz-Rodriguez M and Moyano S (2017) Sepsis: A review of advances in management. Adv Ther 34:2393-2411. 10.1007/s12325-017-0622-8 PMC5702377 29022217

[B24] Rhodes A, Evans LE, Alhazzani W, Levy MM, Antonelli M, Ferrer R, Kumar A, Sevransky JE, Sprung CL, Nunnally ME *et al.* (2017) Surviving sepsis campaign: international guidelines for management of sepsis and septic shock: 2016. Intensive Care Med 43:304-377. 10.1007/s00134-017-4683-6 28101605

[B25] Shen Y, Gong JM, Zhou LL and Sheng JH (2017) miR-451 as a new tumor marker for gastric cancer. Oncotarget 8:56542-56545. 10.18632/oncotarget.17239 PMC5593581 28915610

[B26] Sheng B, Zhao L, Zang X, Zhen J and Chen W (2017) miR-375 ameliorates sepsis by downregulating miR-21 level via inhibiting JAK2-STAT3 signaling. Biomed Pharmacother 86:254-261. 10.1016/j.biopha.2016.11.147 28006751

[B27] Singer M, Deutschman CS, Seymour CW, Shankar-Hari M, Annane D, Bauer M, Bellomo R, Bernard GR, Chiche JD, Coopersmith CM *et al.* (2016) The third international consensus definitions for sepsis and septic shock (Sepsis-3). JAMA 315:801-810. 10.1001/jama.2016.0287 PMC4968574 26903338

[B28] Song P, Shen DF, Meng YY, Kong CY, Zhang X, Yuan YP, Yan L, Tang QZ and Ma ZG (2020) Geniposide protects against sepsis-induced myocardial dysfunction through AMPKα-dependent pathway. Free Radic Biol Med 152:186-196. 10.1016/j.freeradbiomed.2020.02.011 32081748

[B29] Song XW, Zou LL, Cui L, Li SH, Qin YW, Zhao XX and Jing Q (2018) Plasma miR-451 with echocardiography serves as a diagnostic reference for pulmonary hypertension. Acta Pharmacol Sin 39:1208-1216. 10.1038/aps.2018.39 PMC6289348 29795360

[B30] Sun X, Dai G, Yu L, Hu Q, Chen J and Guo W (2018) miR-143-3p inhibits the proliferation, migration and invasion in osteosarcoma by targeting FOSL2. Sci Rep 8:606. 10.1038/s41598-017-18739-3 PMC5766605 29330462

[B31] Verdonk F, Blet A and Mebazaa A (2017) The new sepsis definition: limitations and contribution to research and diagnosis of sepsis. Curr Opin Anaesthesiol 30:200-204. 10.1097/ACO.0000000000000446 28207566

[B32] Wang H, Bei Y, Shen S, Huang P, Shi J, Zhang J, Sun Q, Chen Y, Yang Y, Xu T *et al.* (2016) miR-21-3p controls sepsis-associated cardiac dysfunction via regulating SORBS2. J Mol Cell Cardiol 94:43-53. 10.1016/j.yjmcc.2016.03.014 27033308

[B33] Wang S, Wang Y, Zhang Z, Liu Q and Gu J (2017) Cardioprotective effects of fibroblast growth factor 21 against doxorubicin-induced toxicity via the SIRT1/LKB1/AMPK pathway. Cell Death Dis 8:e3018. 10.1038/cddis.2017.410 PMC5596591 28837153

[B34] Wang X, Wang X, Liu X, Wang X, Xu J, Hou S, Zhang X and Ding Y (2015) miR-15a/16 are upregulated in the serum of neonatal sepsis patients and inhibit the LPS-induced inflammatory pathway. Int J Clin Exp Med 8:5683-5690. PMC4483976 26131152

[B35] Wang X, Zhu H, Zhang X, Liu Y, Chen J, Medvedovic M, Li H, Weiss MJ, Ren X and Fan GC (2012) Loss of the miR-144/451 cluster impairs ischaemic preconditioning-mediated cardioprotection by targeting Rac-1. Cardiovasc Res 94:379-390. 10.1093/cvr/cvs096 PMC3331614 22354898

[B36] Weng N, Sun J, Kuang S, Lan H, He Q, Yang H, Zhang L and Xue H (2019) MicroRNA-451 aggravates kainic acid-induced seizure and neuronal apoptosis by targeting GDNF. Curr Neurovasc Res 17:50-57. 10.2174/1567202617666191223150510 31870266

[B37] Wu SC, Yang JC, Rau CS, Chen YC, Lu TH, Lin MW, Tzeng SL, Wu YC, Wu CJ and Hsieh CH (2013) Profiling circulating microRNA expression in experimental sepsis using cecal ligation and puncture. PLoS One 8:e77936. 10.1371/journal.pone.0077936 PMC3813489 24205035

[B38] Yang AP, Liu J, Yue LH, Wang HQ, Yang WJ and Yang GH (2016) Neutrophil CD64 combined with PCT, CRP and WBC improves the sensitivity for the early diagnosis of neonatal sepsis. Clin Chem Lab Med 54:345-351. 10.1515/cclm-2015-0277 26351925

[B39] Yao Y, Sun F and Lei M (2018) miR-25 inhibits sepsis-induced cardiomyocyte apoptosis by targetting PTEN. Biosci Rep 38:BSR20171511. 10.1042/BSR20171511 PMC5897747 29440462

[B40] Zheng Z, Ma H, Zhang X, Tu F, Wang X, Ha T, Fan M, Liu L, Xu J, Yu K *et al.* (2017) Enhanced glycolytic metabolism contributes to cardiac dysfunction in polymicrobial sepsis. J Infect Dis 215:1396-1406. 10.1093/infdis/jix138 PMC5451607 28368517

[B41] Zhou Y, Song Y, Shaikh Z, Li H, Zhang H, Caudle Y, Zheng S, Yan H, Hu D, Stuart C *et al.* (2017) MicroRNA-155 attenuates late sepsis-induced cardiac dysfunction through JNK and β-arrestin 2. Oncotarget 8:47317-47329. 10.18632/oncotarget.17636 PMC5564567 28525390

